# Eligibility criteria for intraoperative radiotherapy for breast cancer: study employing 12,025 patients treated in two cohorts

**DOI:** 10.1186/1471-2407-14-868

**Published:** 2014-11-24

**Authors:** Amira Ziouèche-Mottet, Gilles Houvenaeghel, Jean Marc Classe, Jean Rémi Garbay, Sylvia Giard, Hélène Charitansky, Monique Cohen, Catherine Belichard, Christelle Faure, Elisabeth Chéreau Ewald, Delphine Hudry, Pierre Azuar, Richard Villet, Pierre Gimbergues, Christine Tunon de Lara, Agnès Tallet, Marie Bannier, Mathieu Minsat, Eric Lambaudie, Michel Resbeut

**Affiliations:** Department of Radiotherapy, Institut Paoli Calmettes, Marseille and CRCM France, 232 Boulevard de Sainte-Marguerite, 13009 Marseille, France; Department of Surgery, Institut René Gauducheau, Nantes, France; Department of Surgery, Institut Gustave Roussy, Villejuif, France; Department of Surgery, Centre Oscar Lambret, Lille, France; Department of Surgery, Centre Claudius Regaud, Toulouse, France; Department of Surgery, Centre René Huguenin, Saint Cloud, France; Department of Surgery, Centre Léon Bérard, Lyon, France; Department of Surgery, Hôpital Tenon, Paris, France; Department of Surgery, Centre Georges François Leclerc, Dijon, France; Department of Surgery, Hôpital de Grasse, Grasse, France; Department of Surgery, Hôpital des Diaconnesses, Paris, France; Department of Surgery, Centre Jean Perrin, Clermont Ferrand, France; Department of Surgery, Institut Bergonié, Bordeaux, France; Aix Marseille Université, Marseille, France; Department of Surgery, Institut Paoli Calmettes, Marseille and CRCM France, 232 Boulevard de Sainte-Marguerite, 13009 Marseille, France

**Keywords:** Breast cancer, Intraoperative radiotherapy, Intrabeam®

## Abstract

**Background:**

We wished to estimate the proportion of patients with breast cancer eligible for an exclusive targeted intraoperative radiotherapy (TARGIT) and to evaluate their survival without local recurrence.

**Methods:**

We undertook a retrospective study examining two cohorts. The first cohort was multicentric (G3S) and contained 7580 patients. The second cohort was monocentric (cohort 2) comprising 4445 patients. All patients underwent conservative surgery followed by external radiotherapy for invasive breast cancer (T0–T3, N0–N1) between 1980 and 2005. Within each cohort, two groups were isolated according to the inclusion criteria of the TARGIT A study (T group) and RIOP trial (R group).

In the multicentric cohort (G3S) eligible patients for TARGIT A and RIOP trials were T1E and R1E subgroups, respectively. In cohort number 2, the corresponding subgroups were T2E and R2E. Similarly, non-eligible patients were T1nE, R1nE and T2nE, and R2nE.

The eligible groups in the TARGIT A study that were not eligible in the RIOP trial (TE–RE) were also studied. The proportion of patients eligible for TARGIT was calculated according to the criteria of each study. A comparison was made of the 5-year survival without local or locoregional recurrence between the TE *versus* TnE, RE *versus* RnE, and RE *versus* (TE–RE) groups.

**Results:**

In G3S and cohort 2, the proportion of patients eligible for TARGIT was, respectively, 53.2% and 33.9% according the criteria of the TARGIT A study, and 21% and 8% according the criteria of the RIOP trial. Survival without five-year locoregional recurrence was significantly different between T1E and T1nE groups (97.6% *versus* 97% [log rank =0.009]), R1E and R1nE groups (98% *versus* 97.1% [log rank =0.011]), T2E and T2nE groups (96.6% *versus* 93.1% [log rank <0. 0001]) and R2E and R2nE groups (98.6% *versus* 94% [log rank =0.001]). In both cohorts, no significant difference was found between RE and (TE–RE) groups.

**Conclusions:**

Almost 50% of T0-2 N0 patients could be eligible for TARGIT.

## Background

Breast cancer (BC) is the leading cancer worldwide in terms of incidence, with 1.38 million new cases diagnosed in 2008 (23% of all cancers). It is now the most frequent cancer in “developed” and “developing” countries [1]. BC management, therefore, is a major issue in terms of public health at therapeutic and economic levels.

Screening by mammography is undertaken in most developed countries. It has enabled BC to be diagnosed at an early stage, thereby allowing the possibility of conservative surgery. Several studies have shown that in conservative surgery of an invasive cancer, adjuvant radiation of the breast and surgical site significantly increases survival without recurrence and has an impact on overall survival [2].

The correlation between local recurrence and metastasis has also been confirmed for tumors treated at very limited stages, at which point it is possible to use partial radiation [3]. However, radiotherapy is not readily available in many countries. Almost 90% of recurrences of BC are localized in the same quadrant [4]. Based on this clinical information and with the objective of making access to radiotherapy easier, partial-breast irradiation methods (e.g., brachytherapy, external radiotherapy, intraoperative radiotherapy) have been developed over the last 20 years.

Here, we describe a retrospective study focusing on patients undergoing conservative treatment associated with partial surgery and conventional external radiotherapy for invasive cancer. We had two main objectives. First, we wished to estimate the proportion of patients eligible for exclusive targeted intraoperative radiotherapy (TARGIT) according to the criteria set by TARGIT A trial and RIOP (Radiothérapie IntraOPératoire) trial (RIOP is a trial being carried out in France, coordinated by the Institut René Gauducheau and it is being conducted under the aegis of the Institut National du Cancer)[5]. Second, we wished to evaluate and compare survival without local recurrence or locoregional recurrence among patients eligible and ineligible for TARGIT according to the criteria of the two trials mentioned above.

## Methods

This was a retrospective study examining two cohorts of patients who had undergone conservative surgery followed by external radiotherapy for invasive, non-metastatic and non-inflammatory BC.

The first cohort was multicentric (cohort G3S) and comprised 7580 patients treated in 13 French centers from 1999 to 2008. All of these subjects had undergone biopsy of a sentinel node with or without axillary dissection for tumors of stages T0–T2 and N0. Characteristics of patients with small tumors and results of treatment are described in a study that will be published shortly [6]. The second cohort was monocentric (cohort 2). It consisted of 4445 patients treated at the Institut Paoli Calmettes (IPC) from 1980 to 2005 by conservative surgery for tumors of stage T0–T3, N0 or N1 with biopsy of the sentinel node associated or not with axillary dissection, or alternatively with immediate axillary dissection.

This study was approved by the ethics committee of the Paoli-Calmettes Institute.

### Anatomopathologic information

Anatomopathologic analyses of each resected specimen defined the size, histologic type and the stage (using the Scarff–Bloom–Richardson classification [SBR]) of the tumor [7], as well as the presence of peri-tumor vascular emboli and nodal invasion. Immunohistochemical methods were used to establish the presence of hormone receptors with estrogens and progesterone at a threshold of 10%. Data relating to overexpression of the human epidermal growth factor receptor (HER2) were too recent compared with the period of the study and insufficiently exhaustive, so they were not used in cohort 2. The overexpressed status (or otherwise) of HER2 was defined in 4732 patients of the G3S cohort.

### Adjuvant therapies

All patients had undergone external radiotherapy of the mammary gland, most frequently in conjunction with a boost to the tumor bed. The systemic treatments that were proposed were chemotherapy in accordance with the data of the various centers and hormone therapy for 5 years for hormone-sensitive tumors.

### Study groups and parameters studied

**Figure 1:**
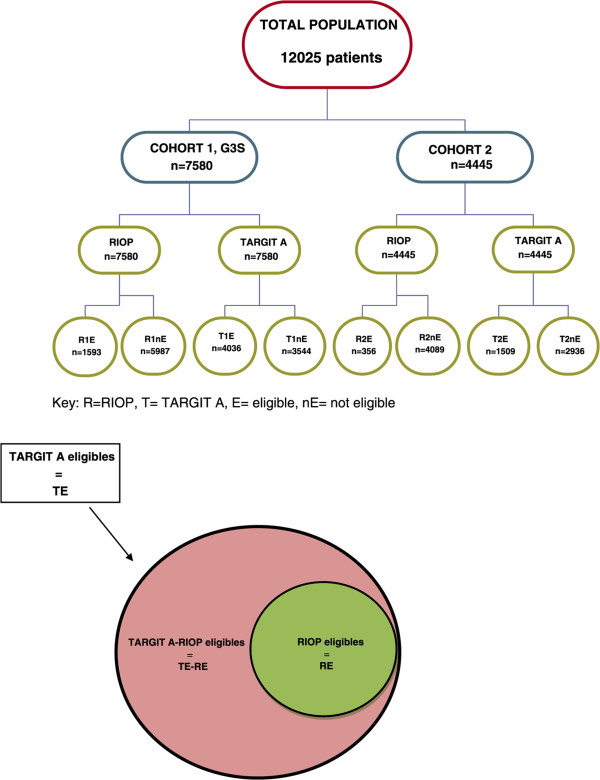
**Groups and sub-groups studied.**

Two groups were isolated within each cohort using the inclusion criteria of the RIOP and TARGIT-A trials. Each group was divided into two subgroups by separating patients who were eligible for TARGIT from those who were not: RIOP trial with subgroups R1E and R1nE for the G3S cohort; R2E and R2nE for cohort 2 and the TARGIT A trial with subgroups T1E and T1nE for the G3S cohort; T2E and T2nE for cohort 2. The subgroup of patients eligible in the TARGIT A study and not eligible in the RIOP study (TE-RE) were also studied in each cohort (Figure 1).

In each trial, eligibility criteria were related to preoperative clinical, radiologic and histologic data. Patients eligible for TARGIT according to the TARGIT A study were aged ≥45 years and had a unifocal lesion of invasive ductal carcinoma. Three postoperative criteria indicated the need for external irradiation of the mammary gland: invasive lobular-type histology; an extensive intraductal component; unhealthy margins (which also suggested the need for further surgical intervention). Grade-3, nodal or lymphovascular invasion could lead to the proposal of external radiation but this decision was left to the discretion of each center. It was preferable that the lesion measured 3.5 cm without this criterion imposing the need for adjuvant external radiation. In the present study, of the 996 randomized patients treated effectively by TARGIT, 142 patients received complementary external radiotherapy without the details of the criterion having indicated this radiation. We considered the following factors as eligibility criteria for TARGIT according to the TARGIT A trial: ≥45 years; unifocal invasive ductal-type lesion; pN0 with healthy excision margins.

The eligibility criteria for TARGIT according to the RIOP trial were: menopausal patients not carrying a known mutation in the breast cancer (BRCA)1 or 2 gene; age ≥55 years; tumor size ≤2 cm; a unifocal invasive ductal-type lesion; tumor grade 1 or 2; expression of hormone receptors; no amplification of HER2 expression; absence of vascular peri-tumor emboli or clinical nodal invasion. Postoperative nodal invasion, an extensive intraductal component, unhealthy excision margins, and failure to comply with the criteria stated above at the preoperative stage rendered complementary external radiotherapy necessary. Inclusion criteria in both studies are summarized in Table 1.

**Table 1:** **Inclusion criteria for TARGIT A and RIOP trials**

	TARGIT A	RIOP
**Age (years)**	≥45	≥55
**Menopausal status**	Not required	Required
**Unifocal tumor**	Required	Required
**Histology**	Invasive ductal carcinoma	Invasive ductal carcinoma
**Tumor size**	Suitable for wide local excision (<3.5 cm preferable)	≤2 cm
**SBR grade 1 and 2, hormone receptor-positive, no vascular invasion, HER2**	Not required	All of these criteria were required
**No known BRCA mutation**	Not required	Required

For each cohort we calculated the proportion of patients eligible for TARGIT according to the criteria of each of the trials. Then, we compared the survival rates without local recurrence or SSRL at 5 years and 7 years of the following groups: RE groups *versus* RnE groups; TE groups *versus* TnE groups; RE groups *versus* (TE–RE).

The diagnosis of local recurrence was defined as appearance of a tumorous lesion developed within the treated breast and confirmed by histologic analyses. Locoregional recurrence comprised axillary local and nodal recurrence, supraclavicular and infraclavicular recurrence, or intra-mammary recurrence. Local recurrence could be identified in cohort 2. Only locoregional recurrence was known in the G3S cohort. However, the literature suggests that nodal recurrences represent a very small number of locoregional recurrences. Thus, locoregional recurrence can be considered to be survival without local recurrence in almost all cases [8–10].

### Statistical analyses

All statistical tests were two-sided. Comparisons were made using: chi-square tests for percentages; Student’s *t*-test for mean values (statistical significance was set at 0.05 for both tests) and log-rank test for survival (statistical significance was set at 0.1). Statistical analyses were carried out using SPSS v16.0 (SPSS, Armonk, NY, USA).

## Results

### Population characteristics

In the total population, mean age at the diagnosis was 58 years (median, 58; range, 22–101) for the G3S cohort and 55.6 years (55; 20–91) years for the IPC cohort. The mean pathologic size of T1 lesions was 15 mm (median, 13; range, 0.1–9.0) and 19.2 mm (17; 1–125) with 84.8% (6425/7580) and 67.7% (3010/4445), respectively, in G3S and IPC cohorts. Within this population, all patients had undergone conservative surgery associated with axillary lymph-node dissection. A total of 36.7% (2784/7580) and 43.6% (1938/4445) of patients received adjuvant chemotherapy, and 84.3% (6373/7556) and 54.6% (2417/4422) of patients underwent adjuvant hormonal therapy for the G3S cohort and cohort 2, respectively. All patients received adjuvant radiotherapy of the mammary gland at 50 Gy, most often with a boost of 16 Gy to the tumor bed.

Characteristics of the eligible patients in the TARGIT A and RIOP studies of each cohort are shown in Table 2. There was a significant difference between the two cohorts in terms of the following characteristics: age; tumor size >20 mm; RH-; pN+; grade; vascular peri-tumor emboli (p < 0.0001). Also, a population showed more unfavorable characteristics in the IPC cohort (p < 0.0001). Table 3 shows that, in each cohort, eligible patients in the RIOP study (R1E and R2E) had significantly different characteristics from the eligible patients in the TARGIT A study and non-eligible patients in the RIOP study (TE–RE).

**Table 2:** **Characteristics of patients eligible for RIOP and TARGIT A trials of the G3S (R1E and T1E), cohort 2(R2E and T2E) cohorts and of the patients included in the TARGIT A trial, TARGIT arm**

		COHORT G3S		COHORT 2		TARGIT A, TARGIT ARM
		R1E	T1E	R2E	T2E	
n		1593/7580 (21%)	4036/7580 (53.2%)	356/4445 (8%)	1509/4445 (33.9%)	996/1113 (89.5%)
Age						
	< 45 years	0	1813 (30.3%)	0/356 (0%)	0/1509 (0%)	17/1113 (2%)
	45-54 years	0	1216 (20.3%)	0/356 (0%)	459/1509 (30.4%)	212/1113 (19%)
	≥55 years	1593 (100%)	2958 (49.4%)	356/356 (100%)	1050/1509 (69.6%)	884/1113 (80%)
Tumor size						
	≤20 mm	1593/1593 (100%)	3674/4036 (91%)	356/356 (100%)	1185/1508 (78.6%)	912/1056 (86%)
	>20 mm	0/1593 (0%)	362/4036 (9%)	0/356 (0%)	323/1508 (21.2%)	144/1056 (14%)
Histology						
	Ductal	1391/1593 (87.3%)	3596/4036 (89.1%)	356/356 (100%)	1509/1509 (100%)	1012/1070 (95%)
	Lobular	0/1593 (0%)	0/4036 (0%)	0/356 (0%)	0/1509 (0%)	47/1070 (4%)
	Mixed	0/1593 (0%)	0/4036 (0%)	0/356 (0%)	0/1509 (0%)	32/1070 (3%)
	Colloids, ductal medullary	202/1593 (12.7%)	440/4036 (10.9%)	0/356 (0%)	0/1509 (0%)	
Grade						
	1	931/1593 (58.4%)	1775/3988 (44%)	211/356 (59.3%)	604/1465 (41.2%)	341/1040 (33%)
	2	662/1593 (41.6%)	1582/3988 (39.2%)	145/356 (40.7%)	620/1465 (42.3%)	540/1040 (52%)
	3	0/1593 (0%)	631/3988 (15.6%)	0/356 (0%)	241/1465 (16.5%)	159/1040 (15%)
pN						
	0	1593/1593 (100%)	4036/4036 (100%)	356/356 (100%)	1509/1509 (100%)	866/1059 (82%)
	1 -3	0/1593 (0%)	0/4036 (0%)	0/356 (0%)	0/1509 (0%)	155/1059 (15%)
	>3	0/1593 (0%)	0/4036 (0%)	0/356 (0%)	0/1509 (0%)	54/1059 (4%)
RH						
	+	1593/1593 (100%)	3537/4036 (87.6%)	356/356 (100%)	851/1499 (56.8%)	962/1063 (90%)
	-	0/1593 (0%)	499/4036 (12.4%)	0/356 (0%)	648/1499 (43.2%)	101/1063 (10%)
EVPT						
	Absent	1593/1593 (100%)	3056/3389 (90.2%)	356/356 (100%)	1081/1391 (77.7%)	881/1022 (86%)
	Present	0/1593 (0%)	333/3389 (9.8%)	0/356 (0%)	310/1391 (22.3%)	141/1022 (14%)
Excision limits						
	Healthy	1593/1593 (100%)	4036/4036 (100%)	356/356 (100%)	1509/1509 (100%)	970/1072 (90.5%)
	Unhealthy	0/1593 (0%)	0/4036 (0%)	0/356 (0%)	0/1509 (0%)	102/1072 (9.5%)
Multifocality						
	No	1593/1593 (100%)	4036/4036 (100%)	356/356 (100%)	1509/1509 (100%)	
	Yes	0/1593 (0%)	0/4036 (0%)	0/356 (0%)	0/1509 (0%)	
Chemotherapy						
	No	1387/1469 (94.4%)	802/3834 (20.9%)	317/356 (89%)	1146/1509 (76%)	997/1113 (90%)
	Yes	82/1469 (5.6%)	3032/3834 (79.1%)	39/356 (11%)	363/1509 (24%)	116/1113 (10%)
Hormonal therapy						
	Yes	1426/1589 (89.7%)	3290/4026 (81.7%)	356/356 (100%)	851/1499 (56.8%)	727/1113 (65%)
	No	163/1589 (10.3%)	736/4026 (18.3%)	0/356 (0%)	648/1499 (43.2%)	386/1113 (35%)
Radiothery boost to tumor bed						
	Yes			318/355 (89.6%)	1316/1461 (90%)	
	No			37/355 (10.4%)	145/1461 (10%)	
Boost dose						
	<20Gy			305/313 (97.4%)	1156/1298 (89%)	
	≥20Gy			8/313 (2.6%)	142/1298 (11%)

**Table 3:** **Comparison of patients of cohorts G3S (R1E) and cohort 2 (R2E) eligible for the RIOP trial with patients eligible for the TARGIT A study and not eligible for RIOP (TE-RE)**

		G3S COHORT		p	COHORT 2		P
		R1E	T1E-R1E		R2E	T2E-R2E	
n		1593/7580 (21%)	2443/7580 (32.2%)		356/4445 (8%)	1153/4445 (25.9%)	
Age				p < 0.0001			
	< 45 years	0	0		0/356 (0%)	0/1509 (0%)	
	45-54 years	0	1248 (51.1%)		0/356 (0%)	459/1153 (39.8%)	
	≥55 years	1593 (100%)	1195 (48.9%)		356/356 (100%)	694/1153 (60.2%)	
Tumor size				p < 0.0001			p < 0.0001
	≤20 mm	1593/1593 (100%)	2081/2443 (85.2%)		356/356 (100%)	829/1152 (72%)	
	>20 mm	0/1593 (0%)	362/2443 (14.8%)		0/356 (0%)	323/1152 (28%)	
Histology				p = 0.002			
	Ductal	1391/1593 (87.3%)	2205/2443 (90.3%)		356/356 (100%)	1153/1153 (100%)	
	Lobular	0/1593 (0%)	0/2443 (0%)		0/356 (0%)	0/1153 (0%)	
	Mixed	0/1593 (0%)	0/2443 (0%)		0/356 (0%)	0/1153 (0%)	
	Colloids, ductal medullary	202/1593 (12.7%)	238/2443 (9.7%)		0/356 (0%)	0/1509 (0%)	
Grade				p < 0.0001			p < 0.0001
	1	931/1593 (58.4%)	844/2395 (34.5%)		211/356 (59.3%)	393/1109 (35.4%)	
	2	662/1593 (41.6%)	920/2395 (37.7%)		145/356 (40.7%)	475/1109 (42.8%)	
	3	0/1593 (0%)	631/2395 (25.8%)		0/356 (0%)	241/1109 (21.7%)	
pN							
	0	1593/1593 (100%)	2443/2443 (100%)		356/356 (100%)	1153/1153 (100%)	
	1 -3	0/1593 (0%)	0/2443 (0%)		0/356 (0%)	0/1153 (0%)	
	>3	0/1593 (0%)	0/2443 (0%)		0/356 (0%)	0/1153 (0%)	
RH				p < 0.0001			p < 0.0001
	+	1593/1593 (100%)	499/2443 (20.4%)		356/356 (100%)	495/1143 (43.3%)	
	-	0/1593 (0%)	1944/2433 (79.6%)		0/356 (0%)	648/1143 (56.7%)	
EVPT				p < 0.0001			p < 0.0001
	Absent	1593/1593 (100%)	333/1796 (18.5%)		356/356 (100%)	725/1035 (70%)	
	Present	0/1593 (0%)	1463/1796 (81.5%)		0/356 (0%)	310/1035 (30%)	
Excision limits							
	Healthy	1593/1593 (100%)	2443/2443 (100%)		356/356 (100%)	1153/1153 (100%)	
	Unhealthy	0/1593 (0%)	0/2443 (0%)		0/356 (0%)	0/1153 (0%)	
Multifocality							
	No	1593/1593 (100%)	2443/2443 (100%)		356/356 (100%)	1153/1153 (100%)	
	Yes	0/1593 (0%)	0/2443 (0%)		0/356 (0%)	0/1153 (0%)	
Chemotherapy				p < 0.0001			p < 0.0001
	No	1387/1469 (94.4%)	720/2065 (20.4%)		317/356 (89%)	829/1153 (71.9%)	
	Yes	82/1469 (5.6%)	1645/2065 (79.6%)		39/356 (11%)	324/1153 (28.1%)	
HT				p < 0.0001			p < 0.0001
	Yes	1426/1589 (89.7%)	1864/2437 (76.5%)		356/356 (100%)	495/1143 (43.3%)	
	No	163/1589 (10.3%)	573/2437 (23.5%)		0/356 (0%)	648/1143 (56.7%)	
Radiothery boost to tumor bed							NS
	Yes				318/355 (89.6%)	998/1106 (90.2%)	
	No				37/355 (10.4%)	108/1106 (9.2%)	
Boost dose							p < 0.0001
	<20Gy				305/313 (97.4%)	851/985 (86.4%)	
	≥20Gy				8/313 (2.6%)	134/985 (13.6%)	

### Proportion of eligible patients, survival without local recurrence and without locoregional recurrence

In the G3S cohort, the proportion of patients eligible for TARGIT was 21% (1593/7580) and 53.2% (4036/7580), respectively, for the criteria of the RIOP and TARGIT A studies (Table 2). Survival without locoregional recurrence after 5 years and 7 years was 98% and 97.1%, respectively, for the R1E group *versus* 97.1% and 94.8% for the R1nE group; 97.6% and 96.4% for the T1E group *versus* 97% and 94.2% for the T1nE group. There was a significant difference in survival without locoregional recurrence between T1E and T1nE groups (log rank =0.009) and between the R1E and R1nE groups (log rank =0.011). There was no significant difference between the R1E and (T1E–R1E) groups (log rank =0.125) (Table 4).

**Figure 2:**
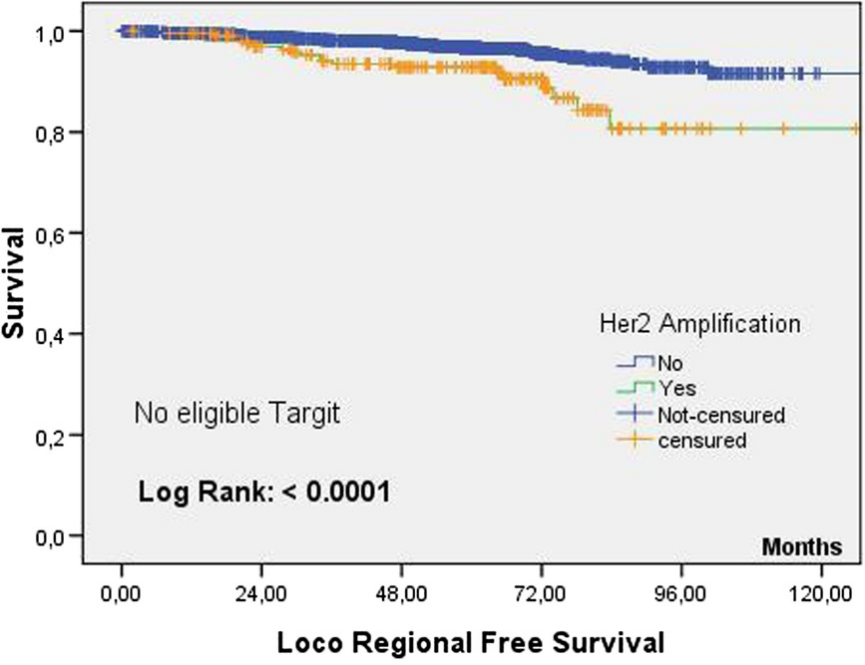
**Survival without locoregional recurrence: patients of the G3S cohort not eligible for the TARGIT A study as a function of the HER2 status.**

In the entire G3S cohort, locoregional recurrences were significantly more frequent in the case of HER2 overexpression (log rank =0.009). Study of subpopulations showed that the eligible patients in TARGIT-A and RIOP studies with tumors overexpressing HER2 did not indicate locoregional recurrence significantly more frequently than that in patients without HER2 overexpression. Conversely, among non-eligible patients with HER2 overexpression, the proportion of locoregional recurrence was significantly greater compared with patients without HER2 overexpression (Figure 2).

**Table 4:** **Comparison of survival without locoregional recurrence of the G3S cohort**

		R1E	R1nE	T1E	T1nE	T1E-R1E
	Number	1589	5982	4031	3540	2442
3 years	%	99.4%	98.3%	98.9%	98.2%	98.6%
	Standard error	0.2	0.2	0.2	0.2	0.3
	exposed to the risk	1347	4554	3109	2792	1761
5 years	%	98%	97.1%	97.6%	97%	97.2%
	Standard error	0.4	0.2	0.3	0.3	0.4
	exposed to the risk	835	2886	1921	1800	1085
7 years	%	97.1%	94.8%	96.4%	94.2%	95.9%
	Standard error	0.6	0.5	0.4	0.6	0.6
	exposed to the risk	243	869	556	556	312
10 years	%	95.5%	91.4%	93.9%	90.6%	92.9%
	Standard error	1.3	1.2	1.4	1.4	2.3
	exposed to the risk	11	27	21	17	9
Log rank			0.011		0.009	0.125*

*R1E vs (T1E-R1E), no significant difference.

Key: RE (eligible for RIOP), R1nE (not eligible for RIOP), T1E (eligible for TARGIT A) T1nE (not eligible for TARGIT A).

*Locoregional recurrence significantly more frequent in the case of over-expression of Her2: only for patients not eligible for TARGIT A and RIOP (selection of the most unfavorable cases).

In the IPC cohort, the proportion of patients eligible for TARGIT was 8% (356/4445) and 33.9% (1509/4445), respectively, for the criteria of the RIOP and TARGIT A trials. Survival without local recurrence after 5 years and 7 years was 98.6% and 97.8%, respectively, for the R2E group *versus* 94% and 90.8% for the R2nE group, and 96.6% and 94.1% for the T2E group *versus* 93.1% and 89.8% for the T2nE group. There was a significant difference in survival without local recurrence between T2E and T2nE groups (log rank =0.0001) and between R2E and R2nE groups (log rank =0.001). There was no significant difference between the R2E and T2E–R2E groups (log rank =0.084) (Table 5).

**Table 5:** **Comparison of survival without local recurrence of the cohort 2**

		R2E	R2nE	T2E	T2nE	T2E-R2E
	Number	356	4089	1509	2936	1153
3 years	%	99.4%	96.3%	98.5%	95.4%	98.3%
	Standard error	0.5	0.3	0.3	0.4	0.4
	exposed to the risk	284	3409	1235	2426	950
5 years	%	98.6%	94%	96.6%	93.1%	96.1%
	Standard error	0.7	0.4	0.5	0.5	0.6
	exposed to the risk	132	2411	804	1714	671
7 years	%	97.8%	90.8%	94.1%	89.8%	93.4%
	Standard error	1.1	0.6	0.8	0.7	0.9
	exposed to the risk	44	1950	465	1113	420
10 years	%	92.6%	87.4%	91.9%	85.9%	91.4%
	Standard error	5.1	7	1.1	0.9	1.2
	exposed to the risk	12	848	221	632	208
12 years	%		83.8%		82.2%	
	Standard error		10		1.2	
	exposed to the risk		539		408	
Log rank			0.001		< 0.0001	0.084*

*R2E vs T2E-R2E, no significant difference.

Key: RE (eligible for RIOP), R2nE (not eligible for RIOP), T2E (eligible for TARGIT A) T2nE (not eligible for TARGIT A).

## Discussion

Standard treatment of early-stage BC is based on conservative surgery followed by radiotherapy. This regimen leads to an increase in survival without recurrence, and in an increase in overall survival if adjuvant radiation is used [2]. Classical adjuvant radiation consists of radiation of the mammary gland followed by a boost to the tumor bed. Depending on the fractionation, this radiation can continue from 3 weeks to 6.5 weeks. Radiotherapy is not available universally (especially in developing countries). Hence, the need to manage patient comfort (not to mention economic considerations), partial and accelerated breast irradiation methods (e.g., brachytherapy, external radiotherapy, intraoperative radiotherapy) have been developed. A reduction in the time between surgery and radiotherapy, and an increase in the dose of equivalent radiation are also possible methods. In addition, radiation (e.g., TARGIT) given during the surgical procedure also seems to act favorably on the tumoral microenvironment [4].

In selected patients, these methods result in a reduction in the volumes treated and duration of radiation without a reduction in survival. In a recent non-inferiority phase-3 randomized trial, Vaidya et al. showed the absence of a significant difference in terms of local recurrence ≤4 years in 2232 randomized patients between external radiotherapy and intraoperative radiotherapy with 50-kV intrabeam photons [5]. Updating of this series has confirmed the efficacy of this treatment over 5 years for patients who have received TARGIT at the time of the initial surgery and not at the time of subsequent treatment [11].

The criteria for inclusion in the TARGIT A trial were deliberately broad to encourage physicians to take part in the trial, giving them freedom in the choice of inclusion criteria and possible complement of external radiotherapy. Nevertheless, results showed homogeneity in the characteristics of patients included suggesting, according to Vaidya et al., conservatism among the physicians participating in that study. Other methods of accelerated partial-breast irradiation using brachytherapy and external radiotherapy for early breast cancer (ELIOT) have confirmed the safety of this type of radiation [12–14]. The difference in the proportion of subjects eligible for TARGIT observed between G3S and IPC cohorts (21% and 8% according to the criteria of the RIOP trial; 53% and 34% according to those of the TARGIT A trial, respectively) can be explained by the significantly greater number of patients with unfavorable tumor characteristics in the IPC cohort. In the G3S cohort, survival without locoregional recurrence at 5 years was 98% and 97.6%, respectively, for patients eligible for TARGIT according to the criteria of the RIOP and TARGIT A trials. In the IPC cohort, survival without local recurrence at 5 years was 98.6% and 96.6%, respectively, for the two groups. Therefore, we would expect comparable values for local recurrence if the patients had been treated effectively by TARGIT.

Although significant, the difference in 5-year locoregional recurrence between T1E and T1nE (97.6% *versus* 97%) was small in absolute terms (0.6%). However, the number of patients still exposed to the risk at 5 years is acceptable (1921/4031 = 46.6% and 1800/3540 = 50.8%, respectively, in T1E and T1NE subgroups).

Wide heterogeneity was present with respect to the indications and procedures of the different treatments. Eligibility criteria for the RIOP trial were much stricter than those of the TARGIT A trial. This difference was why we compared survival without local recurrence or locoregional recurrence of patient group eligible for the RIOP trial with the patients eligible for the TARGIT A trial but not eligible for the RIOP trial (RE *versus* TE-RE): in the two cohorts we observed no significant difference between these groups in terms of survival without local or locoregional recurrence. These results, therefore, encouraged us to extend the eligibility criteria for TARGIT (which were very restrictive in the RIOP trial). Magnetic resonance imaging (MRI) of the breast was not indispensable for inclusion in the present study. Nevertheless, one must question the value of MRI for disqualifying patients with bifocal or multifocal tumors.

HER2 overexpression has been reported to be an aggravating factor for local recurrence, though its value as a factor independent of other criteria (age, emboli, HRs) is controversial [15, 16]. In our study, HER2 overexpression did not aggravate the risk of local recurrence among eligible patients and, in consequence, could not be a factor for exclusion from treatment by TARGIT.

Predictive scores for the risk of local recurrence have been validated in the literature and could be tools for assisting patient selection. In a study by Sanghani et al., significant factors for local recurrence were established by proposing a model starting with patients treated in nine randomized trials. The most significant factors were age ≤40 years (hazard ratio (HR) 2.03), margin invasion (2.19), positive RHs (0.73) and Grade-3 tumors (HR 1.55) [17]. In a study bringing together data from four trials, van Nes et al. proposed a predictive index model with three groups for the risk of local recurrence. However, that study compared local recurrence after conservative surgery and after mastectomy, and did not take account of major forecast-relevant factors such as the SBR grade, RH or vascular peri-tumor emboli [18]. These scores (and others which are still being developed) ought to be able to improve patient selection. However, only a subgroup analysis of randomized trials can inform us if these known “adverse” factors change the effectiveness of radiotherapy.

The potential economic incentive of this method is undeniable because intraoperative radiotherapy provides the best possibility for treatment limited to a single day in an outpatient setting. In our study and according to the subgroups, the outlying percentages of patients who could have benefited from TARGIT were 8% and 53%. Hence, these patients could avoid external radiotherapy or enjoy a reduction in the cost of treatment and the journeys necessary to obtain treatment. In the UK, this treatment would enable the waiting lists for postoperative radiotherapy to be reduced, saving ≈ 23 million dollars. In the United States, IPAS has been proposed as an alternative to standard radiation among patients of the most favorable American Society for Radiotherapy and Oncology (ASTRO) group to limit the time and cost of journeys for postoperative external radiotherapy [19]. The European Society for Radiotherapy and Oncology (ESTRO) also propose a classification that enables selection of patients suitable for partial accelerated irradiation of the breast [20]. A recent study of 59,396 patients showed an increase in the use of partial accelerated irradiation of the breast from 3.4% in 2003 to 12.8% in 2008 (p < 0.001) [21]. Indeed, the ICO 2012–03 Medico-economic Study is underway in France to study the economic incentive of this method.

## Conclusion

TARGIT has potential advantages in terms of cost and efficiency. Patient selection remains an important issue but, given the prevalence of BC, a significant number of treatments could be carried out in this way. Recent data from a randomized trial are encouraging in terms of effectiveness and toxicity, even though a more extensive follow-up program would be indispensable. The results of our study suggest that almost 50% of T0–T2, N0 patients could be eligible for TARGIT with expected survival without local recurrence in 5 years of 96.6–98.6%. Further data from subgroup analyses may further improve patient selection for intraoperative radiotherapy as the only radiation treatment for early BC.

## Consent

Written informed consent was obtained from the patient for the publication of this report.

## Abbreviations

TARGIT: Targeted intraoperative radiotherapy
E: Eligible
nE: Non-eligible
IPC: Institut Paoli Calmettes
RH: Receptor hormone.
